# Unravelling the Intricate Mechanism of Cucurbitacin-Mediated Anti-Cancer Therapy

**DOI:** 10.3390/cancers18142319

**Published:** 2026-07-18

**Authors:** Kankipati Sravya, Shinde Kanchan Pramod Sangeeta, Manash Kumar Paul, Subhadip Mukhopadhyay

**Affiliations:** 1 Manipal School of Life Sciences, Manipal Academy of Higher Education (MAHE), Manipal 576104, Karnataka, India; 2 Department of Biotechnology, Manipal School of Life Sciences, Manipal Academy of Higher Education (MAHE), Manipal 576104, Karnataka, India; 3 Department of Radiation Biology and Toxicology, Manipal School of Life Sciences, Manipal Academy of Higher Education (MAHE), Manipal 576104, Karnataka, India

**Keywords:** cucurbitacin, anti-cancer, synergistic effect, chemoresistance, phytomedicine

## Abstract

Cancer continues to be a primary cause of death globally, necessitating the constant development of effective and less toxic therapeutics. Cucurbitacins belong to the tetracyclic triterpenoids found mainly in the Cucurbitaceae family. Cucurbitaceae plants exert various biological activities such as anti-diabetic, anti-cancer and anti-inflammatory properties, which make them beneficial in addressing metabolic disorders. This review focuses on cucurbitacins namely A, B, C, D, E, I, IIa, which have been explored in cancer research. Cucurbitacins suppress tumor progression by activating cell death pathways, including apoptosis, autophagy, pyroptosis and ferroptosis. They are known to target multiple crucial biomolecular key players, such as STAT3, AKT, mTOR, ERK, EGFR and TLR4. Additionally, they disrupt cytoskeletal proteins and inhibit cell proliferation, invasion, migration, angiogenesis, and cell-cycle arrest. Cucurbitacins have been demonstrated to modulate tumor microenvironment, leading to enhanced host immune surveillance that reverses traditional therapy resistance from cisplatin, doxorubicin, and paclitaxel. In this review, we highlight the strong potential of cucurbitacins as anti-cancer agents, either as monotherapy or in combination, for the development of safer, cost-effective drugs with improved patient treatment outcomes.

## Simple Summary

Cucurbitacins are bioactive triterpenoids, exhibiting potent anti-cancer activity. They act on crucial cellular processes such as cell proliferation, metastasis, and programmed cell death, making them efficient across various cancer types. Their ability to regulate several signaling pathways provides an advantage over traditional chemotherapeutics. Furthermore, Cucurbitacins enhance the efficacy of chemotherapeutic drugs by overcoming resistance in cancer cells. In this review, we summarize the application of cucurbitacins to target different physiologically important pathways of cancer proliferation and highlight their translational journey as potential clinical candidates.

## 1. Introduction

Cancer is a deadly disease caused by uncontrolled cell proliferation, resulting in the formation of a tumor nodule that develops ways to nourish itself while evading host surveillance. Cancer can metastasize from its primary site of occurrence, making therapeutic delivery challenging with bouts of recurrence. Cancer is primarily caused by genetic changes or environmental factors that affect normal cells [1]. Despite major advancements in treatment, diagnostic tools, and preventive measures, cancer continues to be a major cause of mortality. The main treatment options for cancer include surgery, chemotherapy, and radiotherapy. The success rate of cancer treatment depends on the type of treatment and stage of the disease [2]. Some of the major limitations of present therapeutic options include drug resistance, lack of cancer epigenetic profiling, difficulty in diagnosing, absence of biomarkers, drug toxicity, and the metastatic nature of cancer cells [3]. These factors further lead to side effects like pain, anemia, nausea, fatigue, and other adverse effects, such as mitigating a steep financial crisis during therapy management. These drawbacks have fueled the exploration of various natural therapeutics, such as phytomedicines, many of which are derived from dietary sources and part of nutritional intake, to treat different cancers [4,5].

Phytomedicine may be broadly defined as a plant-derived substance that can be made available in various forms for potentially curing a diseased condition [6,7]. They may exist in an isolated form or as a mixture of various secondary metabolites, which are used to reduce disease progression and treat the disease. Phytomedicines might also contain vitamins and minerals such as N, K, P, Zn, Fe, Cu, Mn, Ca, Mg and S, which have both preventive and therapeutic effects. Additionally, phytomedicines can be used to treat drug-resistant cancer [4,8]. The prevalence of phytomedicines has been increasing over the years, providing better therapeutic effects and lower toxicity. Currently, half of the available drugs are derived from natural resources [9]. Experimental studies have shown that plant-based drugs such as paclitaxel, vincristine, and vinblastine are used as chemotherapeutic agents for treating various cancers [10,11]. Bioactive agents obtained from plants are readily available and can be a source of novel drugs [12]. The easy availability of phytomedicines makes it cost-effective for chemotherapy, reduces the mortality rate, and improves the quality of patient care [6].

**Figure 1:**
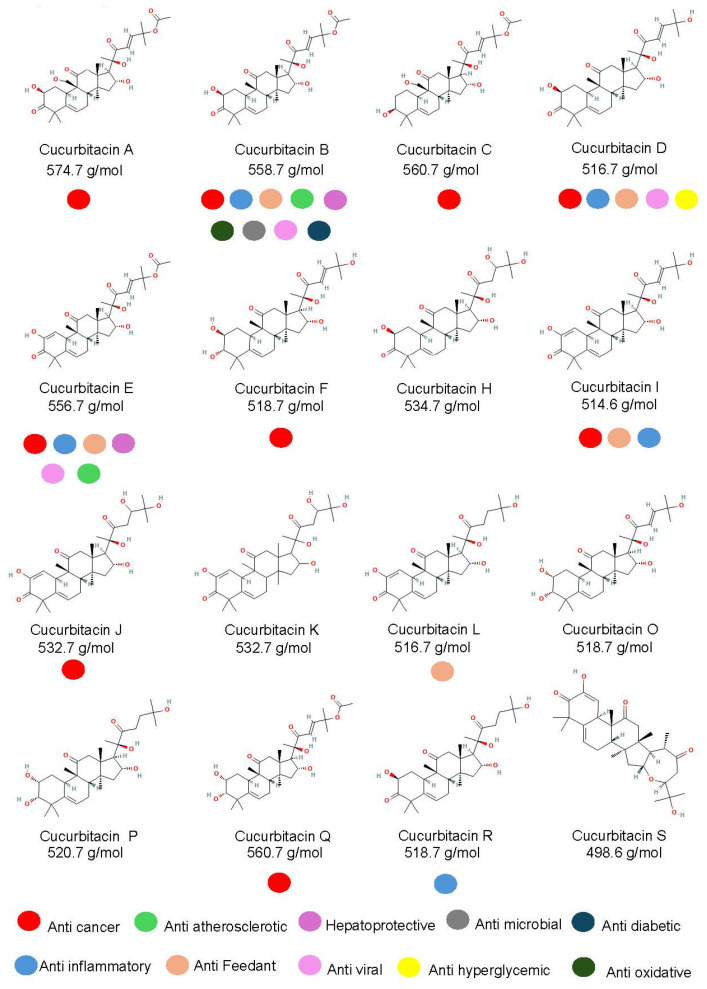
Cucurbitacin chemical structures and their biological relevance are color-coded. Curcubitacins whose biological relevance are yet to be explored were represented without any colored circle.

Cucurbitacins are a group of tetracyclic terpenes that hold great phytomedicinal potential, consisting of many hydroxy, keto, and acetone groups and unsaturated chains. They are mostly found in the Cucurbitaceae family and in many other families like Liliaceae, Rosaceae, Tropaeolaceae and Primulaceae [13,14]. Cucurbitaceae is a large plant family consisting of about 126 genera and 960 species, including cucumbers, gourds, and pumpkins. They are a good source of glucose, fructose, amino acids, vitamins, terpenoids, glycosides, flavonoids, and minerals [15]. Cucurbitacin exhibits many biological effects, such as hepatoprotective, anti-inflammatory and anti-cancer properties. However, the biological activities of some structurally identified cucurbitacin derivatives such as Cucurbitacin H, Cucurbitacin P, Cucurbitacin K, and Cucurbitacin S remain insufficiently explored (shown in Figure 1). They can reduce tumor cell entry, migration and cell death by activating apoptosis, autophagy pathways and cell-cycle arrest. Cucurbitacins have been classified into 12 classes and named from A to T, with more than 200 derivatives based on their structural properties [16]. Previous structure–activity relationship studies of five cucurbitacin molecules, Cucurbitacin A (CuA), Cucurbitacin B (CuB), Cucurbitacin E (CuE), Cucurbitacin I (CuI), and Cucurbitacin Q, have suggested that structural differences can influence target binding. These reports revealed that the conversion of a C3 carbonyl to a hydroxyl group resulted in the loss of its ability to inhibit Janus kinase 2 (JAK2). Similarly, the addition of hydroxyl groups at C11 can result in the loss of the anti-signal transducer and activator of transcription 3 (STAT3) activity [17]. This review provides an overview of the relationship between cucurbitacin molecules and major signaling pathways such as mitogen-activated protein kinase (MAPK), Phosphoinositide 3-Kinase (PI3K)/Protein Kinase B (AKT)/Mammalian Target of Rapamycin (mTOR), JAK/STAT3, Extracellular signal Regulated Kinase (ERK) and Nuclear factor kappa-light chain enhancer of activated B cells (NF-kB) involved in various cancers via major mechanisms involving cell death, cell-cycle arrest and cytoskeleton disruption.

This narrative review summarizes and critically discusses the anti-cancer potential of cucurbitacins and their underlying molecular mechanisms across different cancer types. The relevant literature was collected from electronic databases including PubMed, Scopus, and Science Direct using keywords such as “cucurbitacins,” “cancer,” “apoptosis,” “autophagy,” “ferroptosis,” and “multidrug resistance.” Articles published in English language and relevant to the scope of this review were considered.

## 2. Prevalence of Cucurbitaceae

Cucurbitacins are produced all over the world and have a wide range of uses, including food and beverage products, traditional medicine, and in the cosmetic industry. The majority of plants from the Cucurbitaceae family are edible in the form of either their flesh or as seeds or both [18]. The amount of cucurbitacins differs among the source of tissues. They are mainly concentrated in the fruits and roots of mature plants. The maximum concentration of cucurbitacins is obtained during the maturity [19]. Seeds, leaves, fruits and vegetables from the Cucurbitaceae family exhibit many health benefits and have a positive effect on human health [20]. The plants have medicinal properties and are used as traditional medicine for various disorders, especially in Chinese and Ayurvedic medicines. Cucurbits are also used in the cosmetic industry as an ingredient in many natural soaps. They are frequently added to skin products for their healing, cooling and soothing properties [21]. In ancient times, plants of the Cucurbitaceae family were used as medicine for treating urinary ailments, tumors, diarrhea, skin allergies, kidney stones and high blood pressure. The ideal use of cucurbits in the diet has been shown to reduce oxidative damage and the risk of related disorders [22].

## 3. Role of Cucurbitacin in Various Cancers

**Figure 2:**
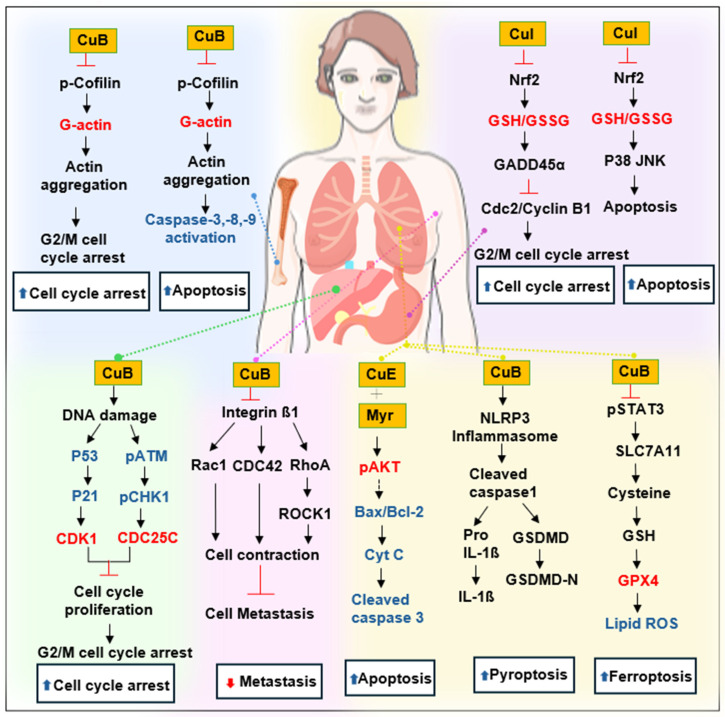
Anti-cancer mechanisms of cucurbitacin in multiple cancers. Cucurbitacins regulated cytoskeletal organization, redox balance, and key signaling pathways to induce G2/M cell-cycle arrest, apoptosis, pyroptosis, and ferroptosis. They aid in DNA damage, mitochondrial dysfunction, caspase activation, inflammasome signaling, and reactive oxygen species (ROS) accumulation, while inhibiting survival, metastatic, and antioxidant pathways. Dotted lines have been drawn to indicated organs which represent the different types of mechanism of action by the reported curcubitacins. For ease of understanding the organ, dotted lines have been color matched and they represent different cancers: blue, leukemia; purple, gastric cancer; green, liver cancer; pink, breast cancer; and yellow, lung cancer. Text shown in blue indicates upregulation/activation, whereas text shown in red indicates the downregulation of signaling molecules. Overall, these findings highlight the broad-spectrum anti-cancer potential of cucurbitacins across multiple cancer types.

**Figure 3:**
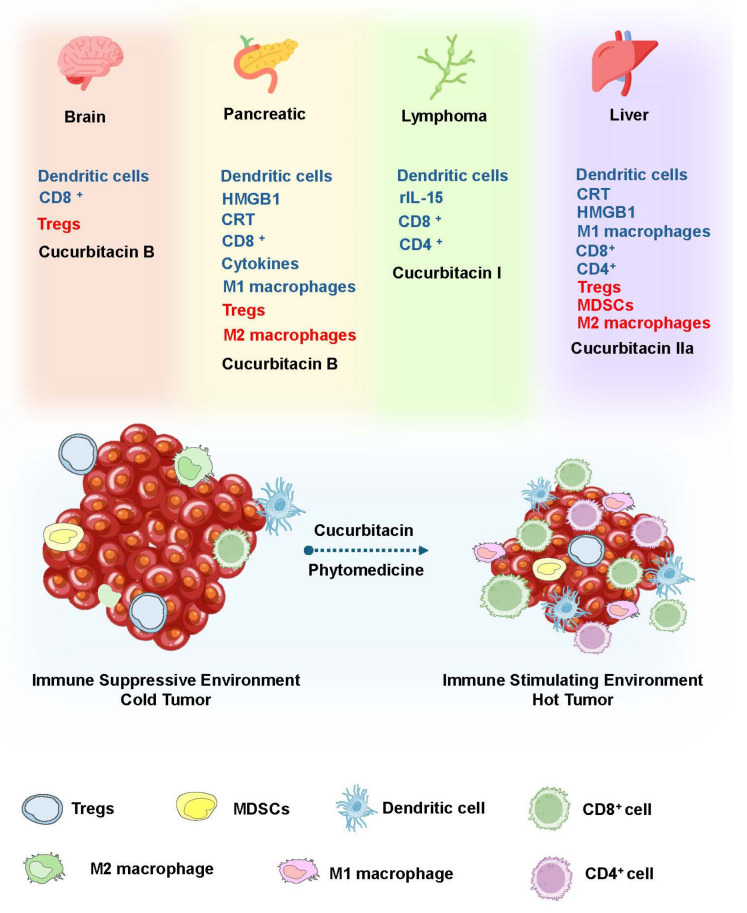
Schematic representation of immune cell infiltration by cucurbitacins in tumor. Cancer cells maintain an immunosuppressive TME, enhanced by regulatory T cells (Tregs), M2 macrophages, and myeloid derived suppressor cells (MDSCs), promoting tumor proliferation. CD8^+^ and CD4^+^ T cells inhibit cancer growth, repress immunosuppressive cells, and regain an anti-tumor environment. Cucurbitacins B, I, and IIa exert anti-tumor activity against brain, pancreas, lymphoma, and liver cells by activating dendritic cells and enhancing immune cell infiltration, ultimately causing immunogenic cell death. Text shown in blue indicates upregulation/activation, whereas text shown in red indicates downregulation.

Cucurbitacins have demonstrated significant anti-cancer potential against a wide range of malignancies, including lung, gastric, prostate, ovarian, liver, breast, colorectal, pancreatic, lymphoma, leukemia, melanoma, glioblastoma, osteosarcoma and oral cancer. Their anti-tumor activities are primarily mediated through the regulation of cell proliferation, apoptosis, autophagy, metastasis, cell-cycle arrest, and multidrug resistance via the modulation of multiple oncogenic signaling pathways (Figure 2). In addition, cucurbitacins influence the tumor microenvironment (TME) by regulating inflammatory mediators, immune cell infiltration, and immune-associated signaling pathways, thereby contributing to anti-tumor immune responses (Figure 3). Furthermore, combinatorial studies with conventional chemotherapeutic agents have demonstrated enhanced anti-cancer efficacy and chemosensitization effects (Table 1), while several studies have highlighted the potential of cucurbitacins in overcoming multidrug resistance in cancer cells (Table 2).

### 3.1. Lung Cancer

Lung cancer is one of the major causes of cancer mortality worldwide. Its high incidence and difficulty in diagnosis make it a crucial issue [42]. As per 2022 statistics lung cancer results in 1.8 million deaths annually [43]. Lung cancer is mainly classified into small-cell lung cancer (SCLC) and non-small cell lung cancer (NSCLC), with NSCLC accounting for 85% of cases [44]. CuA induces morphological changes, including membrane blebbing and nuclear condensation in the human lung carcinoma cell line adenocarcinomic human alveolar basal epithelial cells (A549). It inhibited cancer proliferation by promoting cell-cycle arrest at the G2/M phase and apoptosis via the PI3K/AKT/mTOR signaling pathway. The cells treated with CuA downregulated PI3K, AKT, and mTOR proteins, which are involved in cell survival. The downregulation of these proteins leads to G2/M cell-cycle arrest, further leading to apoptosis and inhibiting cancer growth [45]. CuB shows anti-tumor effect in NSCLC cells via ferroptosis and pyroptosis pathways. CuB treatment in NSCLC cells leads to the accumulation of iron levels while reducing glutathione (GSH) levels and triggers ROS-induced lipid peroxidation that ultimately leads to ferroptosis [46]. In another study, CuB induced ferroptosis by inhibiting STAT3 phosphorylation [47]. This results in an increase in iron and lipid ROS. Inhibition of pSTAT3 also reduces solute carrier family 7 member 11 (SLC7A11) levels, resulting in the downregulation of GSH and glutathione peroxidase 4 (GPX4). This results in the accumulation in ROS in A549 cells and ultimately causes ferroptosis. A study by Yuan et al. reported that CuB inhibits NSCLC in vitro and in vivo by disrupting the Toll-like receptor 4 (TLR4)/NLR Family Pyrin Domain Containing 3 (NLRP3)/Gasdermin D (GSDMD) pathway that leads to pyroptosis [48]. CuB was shown to directly interact with TLR4, resulting in the activation of the NLRP3 inflammasome. Activated NLRP3 separates the GSDMD terminals into N and C terminals. N terminals lead to the formation of pores, causing the release of inflammatory substances, further causing pyroptosis death [49]. NSCLC cells treated with CuB showed increased levels of GSDMD, cleaved caspase 1 and cleaved Interleukin-1ß, suggesting pyroptosis in cells. Again, the in vivo results suggested the inhibition of tumor progression through pyroptosis with no side effects and no decrease in body weight [48]. These findings imply a unique role of CuB in inducing non-apoptotic cell death in NSCLC, unlike CuA, which induced apoptosis by modulating PI3K/AKT/mTOR pathway. CuE has been reported as a tyrosine kinase inhibitor (TKI) that causes STAT3-dependent apoptosis and cell-cycle arrest by hindering the epidermal growth factor receptor (EGFR)/mitogen-activated protein kinase (MAPK) pathway in the A549 cell line. CuE-treated cells showed decreased pSTAT3 levels at low concentration, inhibiting the cell survival pathway and blocking survivin, an apoptosis inhibitor protein [13]. Results demonstrated the evidence of cell-cycle arrest at the G1/G0 phase. CuE-treated cells interfere with members of the EGFR/MAPK signaling pathway. Phosphorylation of mitogen-activated protein kinase kinase 1 and 2 (MEK1/2) was downregulated, and ERK activation resulted in the production of caspase-9 and cyclin B1, which are responsible for apoptosis and cell-cycle arrest [13]. Another study combined CuE with Myricetin (Myr) from *Citrullus colocynthis* to treat NSCLC. The combinatorial therapy CuE and Myr (CuMy-12) inhibited autophagy by reducing Beclin1 levels and upregulating levels of microtubule-associated protein 1 light chain 3 II/chain 3 I (LC3II/LC3I) and p62. CuMy-12 induced cell-cycle arrest in A549 cells by reducing the expression of cyclin D1 and cyclin-dependent kinase 2 (CDK2) and elevating the B cell 2-associated X protein/B cell lymphoma 2 (Bax/Bcl2) ratio, which further led to an increase in cleaved caspase 3 and cytochrome c levels, indicating that CuMy-12 causes apoptosis in A549 cells. This suggests that the combination of CuE and Myr inhibits autophagy and induces apoptosis. CuMy-12 also altered the protein levels of the PI3K/AKT/mTOR pathway [24]. CuI is shown to induce autophagy and apoptosis in A549 cells by inhibiting the ERK/mTOR/STAT3 pathway. Suppression of autophagy by 3-methyladenine treatment could rescue the autophagy induction by CuI and inhibited apoptosis in A549 cells [50]. Interestingly, this study demonstrated autophagy-induced apoptosis, whereas in the previous study, CuE in combination with Myr inhibits autophagy and promoted apoptotic cell death. These contradictory findings highlight the dual role of autophagy as cytoprotective or as cytotoxic depending on the cucurbitacin involved. Cucurbitacin IIa (CuIIa) targets the EGFR-MAPK pathway, inducing cell-cycle arrest, apoptosis, and the suppression of the proliferation of A549 cells. CuIIa on EGFR results in downregulated, phosphorylated, and rapidly accelerated fibrosarcoma 1 (RAF1) and MEK1 and upregulated B-raf proto-oncogene, serine/threonine kinase. Furthermore, the altered EGFR leads to the dysregulation of STAT3 and survivin, causing apoptosis. Cyclin B1 levels decreased, confirming cell-cycle arrest at the G2/M phase. Additionally, CuIIa was proven to be a TKI of the EGFR pathway, confirming its anti-tumor effect on cancerous cells [51]. Similarly to CuE, CuIIa inhibited the same pathway, indicating that different cucurbitacin derivatives share a common mechanism of action with differences in downstream cellular responses.

### 3.2. Gastric Cancer

Gastric cancer ranks as the fifth-most common cancer, with approximately 0.9 million new cases registered, and is the fifth leading cause of death, with approximately 0.8 million deaths worldwide [43]. The incidence of gastric cancer has declined over the decades. Risk factors include genetic syndromes, age, dietary factors and *Helicobacter pylori* infection [52]. CuB has been demonstrated to be an STAT3 inhibitor and that induces apoptosis in gastric cancer cells. In a study by Xu et al., human gastric cancer cell lines were treated with nanomolar concentrations of CuB [53]. The results showed a decrease in the phosphorylation of TYR-705 in STAT3 which further led to abrogation in the levels of cellular myelocytomatosis oncogene (Myc) and B cell lymphoma extra-large (Bcl-xl) anti-apoptotic proteins. Furthermore, CuB-treated cells activated the intrinsic apoptosis pathway by activating caspase 9, followed by caspase 3 and poly (ADP-ribose) polymerase (PARP). Treatment of CuB in mice bearing xenografted gastric tumor showed decrease in tumor [53]. Like lung cancer, in which CuB inhibition of STAT3 results in the activation of ferroptosis in A549 cells, CuB was shown to suppress STAT3 in gastric cancer cells, resulting in the induction of apoptosis, indicating that CuB can induce multiple forms of programmed cell death. CuB triggered the cancerous inhibitor of protein phosphatase 2A (CIP2A)/protein phosphatase 2a (PP2A)/mammalian target of the rapamycin complex 1 (mTORC1) signaling pathway in human cisplatin-resistant gastric cancer cells by inducing apoptosis and autophagy. The mTORC1 is involved in cell growth and metabolism. mTORC1 inhibition activates autophagy and plays an important role in drug resistance. mTOR regulates cellular proliferation by controlling the AKT pathway. CIP2A is an oncogenic protein that is overproduced in tumor cells, where it activates AKT by inhibiting PP2A [54]. CIP2A associates with mTORC1 and activates the pathway promoting cancer progression [55]. Interestingly, Liu et al. demonstrated that CuB induced autophagy and apoptosis in gastric cells by inhibiting CIP2A [37]. The cells resistant to cisplatin were treated with CuB. The results showed a decrease in phosphorylation levels of AKT and an upregulation of PP2A activity. This suggests that CuB targets CIP2A to reduce AKT levels, activate PP2A, inhibit the mTORC1 pathway, and promote apoptosis and autophagy. The depletion of CIP2A also reversed the drug resistance effect. Western blotting confirmed that CuB induced the intrinsic apoptotic pathway. The results showed an increase in PARP cleavage along with an increase in beclin and LC3-II levels, indicating the initiation of the apoptosis and autophagy pathway [37]. Cucurbitacin D (CuD) reduces gastric cancer survival by inhibiting the AKT signaling pathway via the activation of the inducible nitric oxide synthase (iNOS)/nitric oxide (NO). Zhang et al. showed that three human gastric cell lines when treated with different concentrations of CuD led to an increase in Ca^2+^ overload, causing elevated ROS production and changes in mitochondrial membrane potential [56]. Elevated iNOS levels produce NO, which causes mitochondrial apoptosis. This inhibits the AKT pathway. It was also tested in an in vivo model, showing apoptosis and tumor growth arrest [56]. CuE inhibits gastric cancer progression by inhibiting AKT signaling. AKT activation promotes cell survival and proliferation and helps develop resistance to drugs [57]. Si et al. targeted AKT to suppress cancer progression using CuE [23]. In a dose-dependent manner, CuE inhibited AKT signaling, reducing the pAKT levels and leading to G2/M arrest. The results showed an increased number of cells in the G2/M stage. CuE also enhanced the effect of doxorubicin (DOX) by altering AKT levels both in vitro and in vivo. The combination of CuE and DOX leads to cytotoxicity by activating the apoptotic pathway, suggesting a collaborative approach to treat gastric cancer [23]. The results of the above studies reveal that the AKT pathway is one of the common targets of cucurbitacins in gastric cancers. CuB, CuD, and CuE altered AKT levels, resulting in G2/M cell-cycle arrest, apoptosis, and the overcoming of drug resistance.

Deng et al. reported that the low nanomolar concentrations of CuI inhibit the nuclear factor erythroid 2-related factor 2 (Nrf2) and its downstream regulators, which causes redox imbalance [58]. Alterations in redox levels cause increased growth arrest and DNA damage inducible α (GADD45α) protein. Redox imbalance activates c-Jun N-terminal kinase (JNK) and MAPK, activating the GADD45α, promoting G2/M cell-cycle arrest and apoptosis. The same was tested in an in vivo model, CuI-treated cells showed a decrease in tumor weight and confirmed the increase in GADD45α. No side effects were observed, such as toxicity in the organs, impairment in the movement, discomfort, and reduced body weight, suggesting CuI as an effective therapeutic agent against gastric cancer [58].

### 3.3. Prostate Cancer

Prostate cancer is the second leading cancer and a critical issue among men worldwide, accounting for 30% of male cancers in 2025 [59]. Prostate cancer usually occurs in men aged 50 and above. It progresses slowly, with early symptoms being unclear [60]. CuB induces apoptosis and cell-cycle arrest in prostatic carcinoma cells (PC-3) cells by downregulating JAK/STAT signaling. CuB treatment decreased the expression of *JAK1* and *STAT1*, leading to cell death and cell-cycle arrest. It activated caspase-8, 9, and 3, activating both extrinsic and intrinsic apoptotic pathways. CuB also triggers an increase in intracellular ROS and reduces mitochondrial membrane potential, resulting in the onset of apoptosis. CuB exposure reduced the expression of *cyclin D1* and cyclin-dependent kinase 4 (*CDK4*), arresting PC-3 cells at G1/S phase [61]. CuB showed similar effects when induced in the lymph node carcinoma of the prostate (LNCaP) cells by targeting the Notch signaling pathway. Notch signaling plays a crucial role in chemoprotection and anti-cancer effects, which increases the survival of prostate cancer (PCa) cells. Hyperactivated Notch signaling is the characteristic feature of PCa cells [62]. In a study by Alafnan et al., CuB was used to target the Notch pathway [63]. CuB-treated cell downregulated the mRNA expression of *Notch-1*, *jagged-1* and its downstream target hairy and enhancer of split 1 (HES1) protein. Inhibition of the notch signaling pathway leads to a slowdown in progression and an increase in the sensitivity of cells to drugs [63]. Although CuB targets different signaling pathways in PC-3 and LNCaP cells, both investigations demonstrated similar anti-cancer outcomes. This highlights the ability of CuB in modulating multiple pathways, resulting in tumor suppression. CuE triggers apoptosis in human prostate cancer cells, LNCaP, through cofilin 1 and mTORC1. Cofilin 1 is an actin-associated protein present in eukaryotic cells. Cofilin alters the cytoskeleton, resulting in cell–cell adhesion and cell migration. Activated cofilin migrates to mitochondria, triggering mitochondrial fission and activating the intrinsic apoptosis pathway [64]. He et al. reported that CuE increased the expression of cofilin 1 in human prostate cancer cells, suggesting the activation of the mitochondrial apoptosis pathway [65]. CuE-treated cells also exhibited increased mTOR expression. Although mTOR promotes cell survival, the activation of mTOR was inhibited when there was an increase in AMP-activated protein kinase (AMPK) levels. Overexpressed AMPK activated p53, resulting in the release of caspase 9, further promoting apoptosis [65]. Interestingly, cucurbitacins suppress mTOR directly, but in pancreatic cancer, CuE alters mTOR levels through AMPK. These findings suggest that cucurbitacins can influence various pathways through different upstream regulators.

### 3.4. Ovarian Cancer

Ovarian cancer is a common tumor with a high incidence and low cure rate. The prognosis of this disease is inadequate owing to the lack of early diagnosis and potent screening methods [66]. Statistics from 2022 revealed that approximately 0.3 million women were diagnosed with the disease and 0.2 million deaths were reported, marking it as the fourth cause of female cancer death [43]. Findings by Liu et al. concluded that CuA could inhibit ovarian cancer by targeting the mTOR/PI3K/AKT pathway [67]. CuA-treated Sloan Kettering Institute Ovarian 3 (SKOV3) cells showed an increase in cell count at G2/M phase, suggesting that CuA caused cell-cycle arrest at G2/M checkpoint. The results revealed an increase in ROS levels, followed by a reduction in the mitochondrial membrane potential and inhibiting cell migration. The PI3K/AKT/mTOR plays a crucial role in metastasis, proliferation and resistance to therapy. Hence, inhibiting the pathway is crucial for treatment. CuA-treated cells downregulated the mTOR and p-mTOR protein levels and suppressed the PI3K/AKT expression, inhibiting the pathway [67]. Notably, similar effects of CuA have been reported in lung cancer cells, where CuA downregulated PI3K, AKT and mTOR proteins, leading to G2/M cell-cycle arrest and apoptosis. However, in ovarian cancer, CuA downregulated mTOR and inhibited PI3K/AKT, exhibiting the same anti-cancer effects. CuB induces cell-cycle arrest and apoptosis in cisplatin-resistant ovarian cancer by targeting STAT3, NF-kB and MAPK signaling pathways. CuB alone or in combination with cisplatin inhibited the NF-kB pathway, preventing its translocation into the nucleus. Furthermore, it inhibited pSTAT3 levels and activated caspase 3. This resulted in the suppression of PARP-1 cleavage, ultimately leading to apoptosis. CuB, in combination with cisplatin, inhibited the phosphorylation of ERK1/2, causing apoptosis. Additionally, CuB decreases GSH levels, antioxidants, and dual-specificity tyrosine-regulated kinase and increases ROS, resulting in the sensitization of resistant cells to cisplatin [36]. CuB inhibits paclitaxel resistance cancer cells by inhibiting a key multidrug resistance protein 1 (MDR1) protein also known as permeability glycoprotein (P-gp) expression. P-gp resistance and non-pgp resistance are two major causes for multidrug resistance in cancer cells. P-gp overexpression limits drug accumulation and expels them from the cell [68]. CuB inhibited the A2780/Taxol cell by inhibiting P-gp expression, resulting in drug uptake. CuB induced apoptosis by increasing the protein levels of p53, inhibiting anti-apoptotic proteins, and decreasing the levels of pro-caspase 3. The results were confirmed using apoptosis markers such as nuclear fragmentation, chromatin condensation, and apoptotic body formation. This indicates that CuB is a potent therapeutic agent for inhibiting multidrug resistance (MDR) cancer cells [38]. Similar mechanisms of reversing P-gp expression have been reported in liver cancer to overcome drug resistance and induce apoptosis. CuB also inhibits the proliferation of cisplatin-resistant ovarian cancer cells by inducing DNA damage. The findings of this study indicate that CuB induces DNA damage, which is identified by the DNA receptor cyclic GMP-AMP synthase (cGAS). Continuous activation of cGAS by CuB results in DNA damage and the recruitment of the inhibitor of kappa B α (IKBα), eventually targeting the mTOR pathway. This leads to apoptosis, characterized by nuclear condensation, DNA fragmentation and the inhibition of cisplatin-resistant ovarian cancer cells [39]. CuI exhibits an anti-tumor effect in SKOV3 human ovarian cancer cells by inducing apoptosis and altering human epidermal growth factor receptor 2 (HER2) and its downstream targets. HER2 can phosphorylate downstream targets, including PI3K and AKT [69]. In SKOV3 cells treated with CuI, HER2 phosphorylation inhibited forkhead box O3a (FOXO3a) phosphorylation. The findings from the paper suggest that CuI inhibits the HER2/PI3K/AKT/FOXO3a pathway by reducing the phosphorylation levels and inhibiting metastasis and the proliferation of cells. Reduced p-FOXO3a levels result in the activation of caspase 3, leading to apoptosis in cells [70]. A study by Li et al. suggested the induction of apoptosis in ovarian cancer cells by targeting oxidative stress using CuI [71]. The authors showed the p190B- Ras related C3 botulinum toxin substrate 1 (Rac1) signaling axis. SKOV3 cells treated with CuI showed reduced viability and altered Kelch-like ECh-associated protein 1 (Keap1) and Nrf-2 signaling axis, further affecting its downstream target heme oxygenase 1 (*HO-1*), an antioxidant gene. This results in a reduction in antioxidants and an increase in ROS, causing an increase in caspase-3, resulting in apoptosis. CuI was also seen to disrupt the cytoskeleton, resulting in cell shrinkage through the p190B-Rac1 signaling axis [71].

### 3.5. Liver Cancer

Liver cancer is one of the most frequently occurring malignancies with high mortality rates ranking sixth worldwide [43]. CuB inhibits hepatocellular carcinoma (HCC) progression by inducing cell-cycle arrest through DNA damage without causing apoptosis. Li et al. explored ataxia telangiectasia mutated (ATM)-dependent p53-p21-cyclin-dependent kinase 1 (CDK1) and Checkpoint kinase 1 (CHK1)-Cell division cycle 25C (CDC25C) pathways activated by CuB-induced DNA damage [72]. DNA damage detectors such as ATM/ATM and Rad3 related (ATR) are recruited and activated during DNA double strand breaks (DSBs). γ histone variant H2AX (γH2AX) is a marker of DNA damage. It was highly expressed in cells treated with CuB, suggesting that CuB induced DNA damage in vitro and in vivo in HCC. This further led to the activation of p53 and p21, which decreased CDK1 and resulted in G2/M cell-cycle arrest in cancer cells [72]. Activated CHK1/2 phosphorylates CDC25C, leading to cell-cycle arrest at G2/M phase [73]. CuB treatment increased p-CHK1 expression and downregulated CDC25C, leading to cell-cycle arrest. CuB can be a potent therapeutic agent for inducing DNA damage and treating HCC [72,74]. Chen et al. explored the synergistic role of CuB and DOX hydrochloride as a treatment for HCC [26]. Researchers have developed glycyrrhetinic acid (GA)-modified liposomes loaded with DOX and CuB (GA-DOX/CuB-Lips). The developed liposomes exhibited outstanding physicochemical properties, including enhanced target efficiency, cellular uptake and stable drug release. In vitro and in vivo results indicated that GA-DOX/CuB-Lips exerted higher toxicity than DOX/CuB-Lips, indicating a higher target efficiency of GA. This increases ROS levels, leading to a reduction in the mitochondrial membrane potential, ultimately causing caspase-dependent apoptosis. GA-DOX/CuB-Lips could efficiently inhibit metastasis, invasion and angiogenesis. The combination of DOX and CuB demonstrated promising results in the treatment of HCC [26]. Similar chemosensitization effects have been observed with other cucurbitacin molecules in various cancers, suggesting the potential of cucurbitacins in overcoming drug resistance and improving the efficacy of traditional chemotherapeutics such as DOX, cisplatin and paclitaxel. Sun et al. reported a synergistic effect of CuB and curcumin in BEL7402/5-Fluorouracil (5-FU) cells [40]. Combination therapy showed a higher inhibition rate than monotherapy. Curcumin suppressed p-gp expression, reduced mitochondrial potential, and reversed MDR, leading to cell sensitization to 5-FU. This combination significantly decreased mitochondrial potential, suggesting the activation of intrinsic apoptosis. The combination therapy in vivo showed elevated caspase 3 levels and depleted Adenosine triphosphate (ATP) responsible for MDR. These data suggest that CuB and curcumin together are promising targets for treating BEL7402/5-FU cells [40]. CuE suppressed proliferation, invasion, angiogenesis and migration in Huh7 hepatoma carcinoma cells via multiple pathways. The Kyoto Encyclopedia of Genes and Genomics (KEGG) revealed that CuE regulates MAPK, JAK-STAT and cytoskeleton formation to exert its anti-tumor properties. CuE disrupted cytoskeletal organization, leading to the inhibition of cell migration. CuE regulates MAPK targets, and downregulates ERK and p38, whereas it elevates JNK expression, resulting in anti-proliferative and anti-invasive activities. CuE reduces cell viability, leading to cell-cycle arrest at the G2/M phase and increased apoptosis rates by decreasing the phosphorylation levels of the JAK/STAT pathway. These findings suggest that CuE is a potent drug that suppress Huh7 cells by regulating multiple targets [75]. Uremis et al. demonstrated the combined effect of sorafenib and CuE in inducing apoptosis in HCC cells by regulating multiple pathways [27]. The combination of CuE and sorafenib exhibited higher cytotoxicity than monotherapy. This resulted in higher inhibition of cell proliferation, cell-cycle arrest, apoptosis, and DNA damage and reduction in mitochondrial membrane potential levels. Combined treatment decreased pSTAT3, pJAK2, pAKT, and pmTOR levels. It was also found to increase pJAK, p-p38 levels and decrease pERK levels. Additionally, decreased expression levels of *MAPK* genes result in the inhibition of these signaling pathways and the induction of apoptosis in tumor cells [27]. These findings indicate that CuE exerts significant anti-cancer activity both as monotherapy and in combinatorial approaches. CuI was reported to induce cell-cycle arrest in HCC cell lines by regulating the lysine acetyltransferase 2a (KAT2a)/E2 promoter binding factor 1 (E2F1)-Ubiquitin conjugating enzyme E2C (UBE2C) pathway. Previous studies have reported that KAT2a combines with E2F1, resulting in elevated acetylation levels, leading to cell-cycle arrest at the UBE2C promoter site [76]. STAT3 regulates the expression of E2F1, and the inhibition of STAT3 can downregulate UBE2C [77,78]. These findings indicate that CuI inhibits STAT3 expression and alters the KAT2a/E2F1/UBE2C pathway, ultimately causing cell-cycle arrest. Additionally, CuI inhibits the macrophage polarization induced by HepG2 cells [79]. CuIIa in combination with DOX was used to treat liver cancer cells. CuIIa inhibited the proliferation and tumor growth of liver cancer cells in vitro. It caused cell arrest at the G2/M phase and induced apoptosis by elevating caspase 3 levels. CuIIa enhanced the anti-cancer activity of DOX in vitro. Combination therapy ultimately elevated the levels of immune stimulatory cytokines and inhibited the production of immunosuppressive cytokines. Additionally, CuIIa and DOX can induce immunogenic cell death (ICD), as validated by the presence of crucial ICD biomarkers, high mobility group box 1 and calreticulin. It also stimulates the maturation of dendritic cells, activates T helper (CD4^+^ cell), T cytotoxic (CD8^+^ cell), and M1 macrophages. Additionally, ICD suppresses Treg cells, and M2 polarized macrophages and MDSCs, overall maintaining the TME [28]. Üremiş et al. studied the effect of CuD, CuE and CuI on the HepG2 cells to inhibit liver cancer [80]. The findings revealed that these three molecules inhibited cell growth and invasion, leading to cell death via cell-cycle arrest and apoptosis. Increasing dosages of cucurbitacins resulted in cell-cycle arrest at the G2/M phase. Cucurbitacin-treated cells showed a decrease in antioxidants, Bcl-2, and Bcl-xl expression and an increase in oxidants, caspase 3, caspase 9 and Bax levels, ultimately damaging the mitochondria membrane and disrupting redox balance, leading to apoptosis [80].

### 3.6. Breast Cancer

Breast cancer is one of the most commonly occurring cancers in women globally, ranking second in incidence and fourth in mortality. Breast cancer accounted for 2.2 million cases worldwide in 2022 [43]. The risk factors include genetic mutations, lifestyle and reproductive factors. Male breast cancer cases account for 1% among all breast cancer cases [81]. CuB induces DNA damage and autophagy via ROS in Michigan Cancer Foundation 7 (MCF-7) breast cancer cells. CuB-treated cells showed inhibited growth and increased expression of DNA damage markers such as γH2AX, ATM and ATR, indicating that CuB induced DSBs in MCF-7 cells. Furthermore, increased levels of LC3II, Beclin-1 and p-Unc 51-like autophagy-activating kinase 1 and the inhibition of p-mTOR, p-AKT and p62 were observed in cells after treatment with CuB, suggesting that CuB induced autophagy in cells. CuB increases intracellular ROS levels. The results of this study suggest an increase in ROS-mediated DNA damage and autophagy in MCF-7 cells [82]. A similar DNA damage mechanism induced by CuB has been previously observed in liver cancer. These findings highlight ROS-mediated damage as a common anti-cancer effect. In a study published by Bakar et al., CuB in combination with imatinib mesylate (IM) was induced in MCF-7 [25]. CuB-treated cells, either alone or in combination, exhibited reduced cell proliferation and the activation of apoptotic pathways. The combined effect also decreased *MMP-2* gene expression, which plays a vital role in cell invasion and metastasis. Additionally, the synergistic effect of CuB and IM showed an increase in S phase population, followed by a reduction in G2/M phase, suggesting cell-cycle arrest in the S phase. CuB in combination with IM enhances the chemotherapy response in patients. Similar findings have been observed in colorectal cancer (CRC) cells [25]. CuB inhibits metastasis and angiogenesis in breast cancer via the vascular endothelial growth factor (VEGF)/focal adhesion kinase (FAK)/MMP-9 pathway. At low nanomolar concentrations (25nM), CuB inhibited the VEGF/FAK/MMP-9 pathway. CuB binds to VEGF and hinders its interaction with its receptor, vascular endothelial growth factor receptor (VEGFR), resulting in the phosphorylation of FAK and MMP-9, leading to the inhibition of migration, invasion and angiogenesis. Furthermore, at higher concentrations (>100 nm), CuB initiates the formation of apoptotic bodies and represses cell growth. CuB shows dose-dependent effects; at low concentrations, it suppresses metastasis and at higher concentrations induces apoptosis [83]. CuB is shown to target Ras homolog family member A (RhoA) family proteins and alter mechanical, biochemical and cytoskeletal properties. This inhibits the migration and invasion of breast cancer cells. CuB-treated cells downregulated the expression of cytoskeletal proteins, such as F-actin, vimentin, FAK and vinculin, resulting in an imbalance in cytoskeleton organization. This further affects the elasticity and deformability of cells. CuB inhibits integrin ß1 and reduces the expression of Rac1, cell division cycle protein 42 (Cdc42), and its downstream proteins like Wiskott–Aldrich syndrome protein (WASP), WASP family verprolin homologous protein 2 (WAVE2) and actin-related protein 2/3 (ARP2/3) to inhibit cell migration. Additionally, CuB reduces RhoA and Rho-associated, coiled-coil-containing protein kinase 1 (ROCK1) protein levels, altering the cytoskeletal structure and impairing the mechanical properties of cells [84]. CuB induced ferroptosis when combined with erastin, an inducer of ferroptosis. The cells were treated with a combination of CuB and erastin. This combination significantly reduced cell viability and increased cytotoxicity, suggesting the activation of the ferroptosis pathway. Combined therapy showed elevated ROS levels, mitochondrial membrane depolarization, and increased malondialdehyde (MDA) levels along with lower antioxidant levels. These indicated the triggering of typical hallmarks of ferroptosis. They showed rapid iron responsive element binding protein 2 (IREB2) expression and decreased ferroportin 1 expression, which caused an increase in iron and initiated ferritin degradation. Furthermore, it causes a decrease in SLC7A11 expression, reducing antioxidant levels and causing ferroptotic cell death [29]. Ku et al. reported that CuD can induce cell-cycle arrest and apoptosis in DOX-resistant human breast cancer (MCF7/ADR) cells [32]. MCF7 and MCF7/ADR cells were treated with CuD combined with DOX. This combination significantly suppressed the growth of MCF7 cells, whereas CuD reversed the resistance in MCF7/ADR cells. Studies suggest that activated STAT3 is responsible for resistance to DOX in cancer cell lines, suggesting that STAT3 is a marker for drug resistance [85,86]. In a study by Ku et al., STAT3 was highly expressed in MCF7/ADR cells, and the activation of STAT3 was suppressed by CuD through decreasing pSTAT3 levels [32]. CuD inhibited the NF-kB signaling pathway by increasing IkB and NF-kB expressions and reducing pNF-kB expression. Furthermore, CuD suppressed the translocation and transcriptional activity of STAT3 and NF-kB. Obstruction of this pathway leads to apoptosis and cell-cycle arrest. To confirm this, MCF7/ADR cells were treated with CuD and/or DOX. The results showed an increase in sub-G1 and G2/M, indicating cell-cycle arrest at the G2/M phase. CuD-treated cells showed elevated expression of cleaved caspase-3 and cleaved PARP, indicating caspase-dependent apoptosis in MCF7/ADR cells [32].

### 3.7. Colorectal Cancer

CRC is recognized as one of the most prevalent cancers, with approximately 1.9 million new cases, ranking third in incidence and second in deaths [43]. Saglam et al. [31] studied the role of CuB alone and in combination with gefitinib (Gef) in CRC cell lines to induce cell-cycle arrest by downregulating cyclin D1 and apoptosis by targeting the EGFR and JAK/STAT pathway. CuB and/or Gef suppress the pEGFR and pSTAT3 levels, resulting in the inhibition of these pathways. The combined dosage of CuB and Gef resulted in the higher inhibition of cell proliferation, activation of caspase 3, reduction in DNA synthesis and enhanced DNA fragmentation when compared to monotherapy. The findings of this study demonstrated that CuB in combination with Gef resulted in effective treatment against CRC cell lines [31]. CuB and CuI targeted the notch signaling pathway and cancer stem cells (CSCs) to inhibit colon cancer growth. CuB and CuI reduce cyclin B1 and CDK1 levels, resulting in the G2/M cell-cycle arrest of cells. The data confirm that CuB and CuI cause caspase-mediated apoptosis by upregulating Bax and effector caspases. In vitro and in vivo results showed the inhibition of cancer cell growth and stemness by targeting the Notch signaling pathway. Both compounds bind to the ankyrin repeats in the Notch 1 receptor, resulting in the inhibition of the pathway by reducing the expression of Notch receptors, ligands, γ secretase complex and target genes. The repression of stemness was also validated by the reduction in CSC markers. These findings indicate that CuB and CuI are effective therapeutics for inhibiting colon cancer growth [87]. Liu et al. reported seven key target genes through which Cucurbitacin C (CuC) exerts its therapeutic effect on CRC through network pharmacology [88]. MMP-1, MMP-3, MMP-9, MMP-13, placental growth factor (PGF), and CDK2 were found to be crucial biological targets. Studies suggest that MMP and PGF play a vital roles in the invasion, metastasis and angiogenesis of cells in various cancers [89,90]. The therapeutic effect of CuC was assessed, which led to the downregulation of PGF and MMPs, inhibiting cell migration and causing apoptosis [88]. Network pharmacology and molecular docking studies revealed four crucial targets, *STAT3*, *AKT1*, *cyclin D1* and *caspase 3*. CuD targets these components and is effective in treating CRC. Additionally, KEGG analysis reports indicate that CuD can target various pathways like the PI3K-AKT pathway, JAK/STAT pathway and Erythroblastic oncogene B pathway, and exert its anti-tumor potency against CRC. Overall, this study highlights the role of CuD in multiple pathways and targets for CRC treatment [91]. CuE sensitizes CRC cells to 5-FU by targeting the transcription factor AP-4 (TFAP4)/Wnt/ß-Catenin pathway. The report suggests TFAP4 as a potent target for CuE, as TFAP4 overexpression results in the reversal of ß-catenin, ATP-binding cassette subfamily C member 1 (ABCC1) and multidrug resistance protein 1 (MDR1), ultimately leading to tumor progression. The combined treatment of CuE and 5-FU downregulates TFAP4 which eventually decreases ß-Catenin, ABCC1 and MDR1 levels and results in the sensitization of cells to 5-FU. Combinatorial therapy results in the significant repression of tumor growth and boosts the chemosensitivity of drug-resistant cells to 5-FU [34]. The colon cancer cell line COLO205, treated with CuI, showed decrease in cell migration and invasion and sensitized the cancer cells to chemotherapy. In this study by Song et al. [35] CuI was combined with 5-FU to sensitize the colon cells. This combination resulted in enhanced cell death. These results were validated by inhibiting the activation of STAT3 and reducing MMP-9, a crucial enzyme involved in cell invasion. These results suggest CuI as a potent chemotherapeutic [35].

### 3.8. Pancreatic Cancer

Pancreatic cancer is a solid malignant tumor that ranks sixth in terms of mortality rate, with approximately 0.4 million deaths recorded in 2022 [43]. Pancreatic cancer accounts for approximately 5% of all cancer-related deaths globally. Most of the population suffering from pancreatic cancer dies within a year due to limited treatment options, resistance to chemotherapy and poor prognosis [92]. CuB is shown to target pancreatic cancer cells via a triple mechanism. CuB induces mitophagy by inhibiting the PI3K/AKT/mTOR pathway, resulting in the decreased expression of mitochondrial transcription factor A (TFAM), causing mitochondria degradation and the release of mtDNA, thereby activating the PTEN-induced kinase 1/Parkin pathway. These pathways dysregulate energy metabolism and suppress tumor proliferation. CuB also targets LDHA, a crucial glycolytic enzyme. CuB downregulates lactate dehydrogenase A (LDHA), Nicotinamide adenine dinucleotide (NAD^+^)/NADH ratio, oxygen consumption rate and extracellular acidification rate levels, disrupting the glycolytic pathway and growth of cancer cells. Additionally, CuB results in dendritic cell (DC) maturation, an increase in CD8^+^ cells, M1 macrophage and inflammatory cytokines, the downregulation of Tregs, anti-inflammatory cytokines and M2 polarization, suggesting the induction of ICD and improvement in the TME. In conclusion, CuB inhibits cell proliferation, invasion and metastasis by inducing mitophagy, suppressing glycolysis and elevating the immune response [93]. CuB exerts an anti-tumor effect on pancreatic cancer cells alone or in combination with SCH772984, an ERK inhibitor, by targeting the EGFR signaling pathway and its downstream targets. The results demonstrated the downregulation of EGFR, STAT3, AKT and Ribosomal protein S6 and an increase in ERK levels by activating the AMPK pathway in pancreatic cells treated with CuB. Activated ERK plays a vital role in cell growth and in inhibiting apoptosis. Thus, inhibiting ERK is an effective therapeutic target [94]. Furthermore, CuB was combined with the ERK inhibitor SCH772984 to inhibit pERK and activate Bcl-2-mediated apoptosis [30]. Xu et al. [95] reported the role of CuC in inhibiting pancreatic ductal adenocarcinoma (PDAC). CuC-treated cells inhibited the invasion and migration of PDAC cells by inhibiting epithelial–mesenchymal transition. CuC increased cyclin B1 expression, indicating cell-cycle arrest at G2/M phase, followed by an increase in cleaved caspase-3 and cleaved PARP-1, leading to apoptotic cell death. Transcriptomic studies of PDAC cells treated with CuC revealed that cyclic guanosine monophosphate (cGMP)—Protein Kinase G (PKG)-Vasodilator-activated phosphoprotein (VASP) plays a crucial role in inhibiting PDAC cell progression. Reduction in PKG1, Protein Kinase cGMP dependent 2, and p-VASP was observed after CuC treatment [95]. CuI alters the JAK2/STAT3 signaling pathway, which plays an important role in cell proliferation, growth and survival, and is associated with various cancers. CuI-treated cells showed a reduction in pJAK2 and pSTAT3, resulting in a decrease in cell proliferation. Increasing doses of CuI led to a reduction in cell viability, an alteration in morphology, a decrease in mobility and the inhibition of colony and size formation. CuI increased cyclin B1 and decreased cyclin D1 and A2 levels. This results in cell-cycle arrest at G2/M phase. In addition, the induction of apoptosis was confirmed by a reduction in PARP1 and Caspase3. In vivo treatment in mice, showed the same result with no toxicity, as there was no reduction in body weight and any signs of poisoning in the mice, suggesting CuI as an effective therapeutic for inhibiting pancreatic cancer [96].

### 3.9. Lymphoma

Lymphoma is a diverse class of lymphoid cancers that occur in the lymphatic system. They are classified based on the origin of the cell: B cells and T cells. Based on morphology, lymphomas are Hodgkin and non-Hodgkin [97] are present. According to 2022 statistics, non-Hodgkin lymphoma is the 10th most commonly diagnosed cancer, with approximately 0.5 million new cases and is the 11th cause of cancer mortality, with 0.25 million deaths [43]. Ueno et al. [98] reported the role of CuB in inducing apoptosis in primary effusion lymphoma (PEL), which is mostly found in immunosuppressed patients and patients with Acquired Immune Deficiency Syndrome affected by HHV-8/KSHV virus. CuB reduced G-actin levels, causing actin aggregation by decreasing p-cofilin levels in BCBL-1 cells. Damage to the cytoskeletal structure led to the induction of G2/M cell-cycle arrest, further increasing the rate of caspase-mediated apoptosis. Additionally, intraperitoneal injection of CuB into immunocompromised mice suppressed tumors with no toxic effects, suggesting that it is a potent drug to treat PEL [98]. A study combined activated recombinant IL-15 (rIL-15), tumor lysate-pulsed DCs and CuI to effectively treat highly aggressive lymphoma. rIL-15 plays a crucial role in restoring DC function, enhances the infiltration of CD4^+^ and CD8^+^ T cells at tumor sites, and elevates tumor necrosis factor α (TNF-α) and TNF-related apoptosis-inducing ligand expression, resulting in successful tumor death. CuI inhibits the STAT3 survival signaling pathway and sensitizes cells to rIL-15-mediated cytotoxicity, inhibiting metastasis and promoting effective therapy for highly aggressive cancers [99]. Another similar study combines CuI and TNF-α obtained from DCs to treat DOX-resistant lymphoma. Activated rIL-15 produces TNF-α in DCs and enhances its anti-tumor potential against lymphoma. However, its levels decrease as the disease progresses. The findings reported that STAT3 hyperactivation and elevated Bcl-2 are responsible for resistance, proliferation, metastasis and the prolonged survival of cancer cells. CuI suppressed pSTAT3 and sensitized cells to TNF-α-mediated cytotoxicity. CuI in combination with rIL-15 enhances the anti-cancer effect and prolongs the survival rate of mice with resistant lymphomas from 18 days to 35 days and non-resistant lymphomas up to 50 days, and inhibits the migration and growth of cancer cells [41]. CuE and CuI have been shown to induce apoptosis in cutaneous T cell lymphomas (CTCLs) and Sezary cells by hindering the JAK/STAT pathway. CuE and CuI inhibit STAT3, which leads to the induction of apoptosis in CTCLs and inhibits the proliferation of CTCLs and Sezary cells. The reports demonstrate the inhibition of JAK2 by both cucurbitacin molecules and the inhibition of STAT5 only by CuI, which requires further exploration in targeting CTCLs. Overall, targeting JAK/STAT through cucurbitacin molecules can be an effective therapy for CTCLs [100].

### 3.10. Leukemia

Leukemia is characterized by the significant proliferation of unusual white blood cells emerging from the bone marrow [101]. Leukemia ranked 13th with approximately 0.4 million new cases, and 10th as the leading cause of cancer mortality, with approximately 0.3 million deaths in 2022 [43]. CuB showed inhibitory effects in acute myeloid leukemia by targeting CIP2A/PP2A/C-KIT proto-oncogene receptor tyrosine kinase (C-KIT) signaling axis. The findings from the study reveal that CuB downregulates CIP2A to reactivate PP2A expression to inhibit AKT, which plays a crucial role in cell survival. The same CIP2A/PP2A pathway was found earlier in gastric cancer, wherein CuB inhibited the AKT/mTORC1 signaling pathway, induced apoptosis and autophagy, and restored sensitivity to cisplatin. This indicates that targeting the CIP2A/PP2A pathway by CuB is common in various forms of cancers. Furthermore, CuB inhibited C-KIT phosphorylation. CuB also inhibited the phosphorylation of its downstream molecules, JAK2 and STAT3. The results concluded that CuB inhibits cell proliferation and induces caspase-dependent apoptosis by hindering the CIP2A/PP2A/C-KIT signaling axis [102]. A study combined CuI and TNF-α obtained from DCs to treat doxorubicin-resistant leukemia cells. TNF-α is impaired in chronic myeloid leukemia (CML) patient DCs, which results in cell growth and proliferation. CuI reverses TNF-α-mediated cytotoxicity in CML-sensitive patients, resulting in the inhibition of tumor growth. STAT3 is highly active in doxorubicin-resistant leukemia cells, elevating anti-apoptotic proteins and inhibiting apoptosis. Higher doses of CuI partially inhibited STAT3 hyperactivation and weakened the signaling pathway. The study suggests that CuI is an effective therapeutic agent, requiring a more comprehensive approach to effectively treat doxorubicin-resistant leukemia [41]. The sensitization of cancer cells through the inhibition of STAT3 by CuI was similarly noted in leukemia, where the inhibition of STAT3 led to restored TNF-α-induced cytotoxicity, thus minimizing survival signaling.

### 3.11. Melanoma

Melanoma is caused by the uncontrolled proliferation of melanocytes in the epidermis. It is mainly caused by ultraviolet exposure. It accounts for 0.3 million new cases per year, with an annual incidence increase of 1.16% [43,103,104]. CuB exerts anti-melanoma activity by targeting MAPK signaling pathways. CuB-treated cells demonstrated dose-dependent cleavage of initiator and executor caspase and PARP. This further elevated ROS levels and induced apoptosis. These results demonstrate that CuB induces both intrinsic and extrinsic apoptosis in melanoma cells. CuB downregulated the major targets of MAPK, pERK1/2, pMEK1/2 and pSTAT3. The in vivo results exhibited the same pattern of inhibition as the in vitro results, indicating that CuB is a potent drug to inhibit melanoma proliferation [105]. CuB targets the glucose-regulated protein (GRP78)/forkhead box M1 (FOXM1)/kinesin family member 20A (KIF20A) pathway to suppress conjunctival melanoma (CM). The results showed that CuB binds to GRP78 and inhibits the ATPase activity, resulting in pathway inhibition. Additionally, downregulation of GRP78 could lead to alterations in the FOXM1-KIF20A pathway. FOXM1 is a transcription factor that alters the levels of cell-cycle genes such as CDK1 and cyclin B1. FOXM1 also binds to polo-like kinase 1 (PLK1) and controls cell-cycle activity [106]. KIF20A is a downstream target of FOXM1. CuB suppressed KIF20A by inhibiting the FOXM1/PLK1 pathway, which further results in a reduction in Cyclin B1 and CDK1, causing G2/M cell-cycle arrest in CM cells [107]. Liu et al. suggested CuE as a chemotherapeutic to agent prevent the proliferation of melanoma cells in patients with elevated hydroxysteroid dehydrogenase-like 2 (HSDL2) levels [108]. The results reported that HSDL2 is highly present in melanoma cells, responsible for the growth of melanoma cells, and inhibits apoptosis by activating the ERK1/2 and AKT pathways. CuE repressed the ERK and AKT pathways, leading to the downregulation of HSDL2 levels, causing apoptosis and inhibiting cell proliferation in melanoma cells. CuE resulted in the downregulation of Bcl2 and upregulation of Bax and cleaved caspase 3, activating apoptosis and inhibiting tumor growth in a xenograft model [108]. Cucurbitacin E glucoside (CEG) was used to induce apoptosis in A375 melanoma cells. CuE inhibited cell proliferation, increased the G1 population, downregulated cyclin D1, CyclinE2, CDK2, CDK4, reduced antioxidants, and upregulated p53 along with MDA levels, indicating the activation of the apoptosis pathway. Furthermore, it elevated *AMPK* and reduced phosphoglycerate kinase 1 and pyruvate kinase M2 (*PKM2*) in tumor cell pathways. Downregulation of *PKM2* showed obstruction in ATP generation in tumor cells after treatment with CEG, disrupting the metabolic pathway in cancer cells [109].

### 3.12. Glioblastoma

Glioblastoma (GBM) is the most common cancer of the nervous system, accounting for 50% of primary tumors and 20% of intracranial tumors. The survival rate of patients diagnosed with glioblastoma is less than 12 months. The prognosis remains unclear, despite developing therapeutics [110,111]. CuB inhibits glioblastoma via the STAT3/ROS/endoplasmic reticulum stress (ERS) pathway. CuB suppresses cell proliferation and the colony formation of GBM cells, while inducing cell-cycle arrest at the G2/M phase. CuB induces apoptosis by inhibiting pSTAT3, resulting in elevated ROS levels, which further activates ERS. An increase in ERS proteins like eukaryotic translation initiation factor 2α (eIF2α) and C/EBP homologous protein (CHOP) indicates the induction of apoptosis in GBM cells. To enhance drug uptake, a liposome coated with CuB was prepared. The results showed excellent penetration of CuB encapsulated in cancer cell membrane-coated liposomes (M@CuB-Lips) in CL261 cells [112]. Further in vivo results revealed that M@CuB-Lips reduced pSTAT3 levels in tumors. CuB’s effect on the endoplasmic reticulum can induce ICD. This was confirmed by the increased infiltration of CD8^+^ T cells and inhibition of Treg cells in GBM cells, suggesting CuB as an immunotherapeutic drug [112]. CuB exerts anti-tumor property by targeting integrins in glioma cells. CuB interacts with α5ß1 integrin to inhibit the adhesion of glioma cells to fibronectin and laminin-1. Cell migration assays revealed that CuB decreased the motility and velocity of cells towards fibronectin. Cell adhesion, α5ß1 and αv integrins play a crucial role in angiogenesis, and CuB targets these integrins and exhibits anti-angiogenesis activity by blocking in vitro tubulogenesis [113]. CuE induces G2/M arrest by acting on GADD45ß in brain cancer cells. Cheng et al. [114] reported that CuE can inhibit cell proliferation and form the CDK1/GADD45 complex. CuE downregulated cyclin B1 levels, causing displacement from the CDC2/cyclin B1 complex and upregulating GADD45ß, forming the CDK1/GADD45ß complex, which induces cell-cycle arrest at G2/M phase [114]. A similar GADD45-mediated anti-cancer pathway was observed in gastric cancer, where CuI activated GADD45α signaling and induced redox imbalance. These findings suggest an important role of GADD45 family proteins in cucurbitacin-mediated cancer suppression. CuE has also been shown to inhibit glioblastoma cell proliferation via the FAK/AKT/Glycogen synthase kinase 3 ß (GSK3ß) pathway. FAK is phosphorylated and highly expressed in many tumor cells, and is responsible for the migration, growth and proliferation of tumors [115]. GSK3ß is a downstream target of AKT, which is a major signaling pathway responsible for the development of various cancers [116]. CuE downregulated the phosphorylation levels of FAK, AKT and GSK3ß. The findings indicate that CuE blocked the epidermal growth factor-induced phosphorylation of FAK, AKT and GSK3ß, resulting in the obstruction of the proliferation of GBM cells. CuE further decreased the expression of cyclin D1 and Cyclin B1 [117].

### 3.13. Osteosarcoma

Osteosarcoma (OS) is the most prevalent bone malignancy, accounting for 20% of all cases worldwide. OS commonly occurs in long tubular bones like the tibia, femur and humerus [118]. Li et al. [33] developed a nanostructured lipid carrier (NLC) containing methotrexate (MTX) and CuB to treat MTX-resistant OS cells. The efficacy of MTX-CuB-NLC was assessed in vitro and in vivo. The results showed enhanced drug uptake and cytotoxicity against Uppsala 2 OS (U2OS)/MTX cells. The combination of drugs resulted in cell-cycle arrest in the G2 phase and elevated apoptosis levels, detected by flow cytometry. MTX resistance in OS cells occurred due to disruption in the PI3K/mTOR pathway. It has been hypothesized that CuB alters the PI3K/mTOR pathway and reverses MTX resistance, resulting in drug uptake [119]. Further research is required to validate the mechanism behind the reversal of MTX and to determine the ratio for these two drugs [33]. Zhang et al. [120] reported the anti-tumor activity of CuB by downregulating JAK2/STAT3 and MAPK pathways, leading to the inhibition of cell proliferation, cell invasion, cell metastasis and onset of apoptosis. CuB-treated cells downregulated the expression of MAPK proteins such as JNK, p38 and ERK1/2. This led to the onset of apoptosis by modulating Bcl-2 family proteins. Furthermore, the inhibition of the JAK2/STAT3 pathway led to a reduction in the expression of VEGF and MMP. The reduction in VEGF, MMP-2 and MMP-9 levels resulted in the repression of cell invasion and migration and hindered angiogenesis. These results suggest CuB is an effective therapeutic to treat OS, which should be further validated by performing in vivo tests [120]. It is also important to note that, in contrast to gastric cancer, where the activation of JNK is responsible for inducing apoptosis, the downregulation of JNK, p38 and ERK1/2 is associated with the activation of apoptosis in osteosarcoma.

### 3.14. Oral Cancer

Oral cancer, mainly oral squamous cell carcinoma (OSCC), is a prevalent head and neck malignancy that develops in the oral mucosa. OSCC most frequently occurs in males than in females, resulting in difficulty in swallowing, speech and taste [121]. CuB inhibits OSCC and causes T cell infiltration via the STAT3/Caspase-3/Gasdermin E (GSDME) pathway. Bioinformatic studies have revealed the role of pyroptosis in cells, and the results were validated in vivo in a mouse model. The OSCC-induced mice were treated with CuB. The inhibition of STAT3 by CuB led to the activation of Caspase 3, which cleaved the GSDME, resulting in the formation of GSDME-N. This further releases cytokines and causes pyroptosis, with ROS also playing a crucial role in this process. This also leads to T cell infiltration in the tumor region, increasing the chances of survival and showing no side effects to normal organs [122]. Similar results of CuB were found in lung carcinoma, whereby the TLR4/NLRP3/GSDMD pathway was activated by CuB treatment. In NSCLC cells, cleavage of GSDMD into two pieces, N- and C-terminal segments, created pores that initiated cell death via pyroptosis, indicating that CuB can induce gasdermin-dependent pyroptosis in cancer cells. CuB inhibited the migration, entry, and proliferation of tongue squamous cell carcinoma (TSCC) cells and caused apoptosis. Tao et al. conducted a bioinformatics study that revealed the presence of 177 Long noncoding RNAs in CuB-treated cells, with X-inactive-specific transcript (XIST) being the most highly regulated and having an affinity to bind with miR-29b sites, indicating the role of XIST and miR-29b in causing TSCC [123]. CuB downregulated XIST expression, leading to the overexpression of miR-29b, which causes apoptosis through the activation of p53 protein, leading to the inhibition of tumor growth and invasion [123].

## 4. Discussion

Cucurbitacins are highly oxygenated triterpenoids most profusely found in the Cucurbitaceae family, comprising four rings and 30 carbon atoms. They are categorized into 12 classes A to T with more than 200 derivatives based on their structural characteristics. Cucurbitacins from A to IIa have been shown to exert excellent anti-tumor properties across various cancers. Despite the structural similarities and differences among cucurbitacin derivatives, several related mechanisms have been observed. The most common pathways targeted included JAK/STAT3, PI3K/AKT/mTOR, MAPK, EGFR and Notch signaling pathways, which are all essential pathways in the proliferation, survival, metastasis and therapy resistance of cancer cells. Of these pathways, STAT3 appears to be the prime target. STAT3 signaling is inhibited in various cancers, such as lungs, gastric, ovarian, colorectal, leukemia, lymphoma, glioblastoma, melanoma and OSCC, by Cucurbitacin B, I, and E. The dysregulation of STAT3 is correlated with various cellular effects, including apoptosis, ferroptosis, pyroptosis, cell-cycle arrest, and chemoresistance reversal. The recurrent targeting of STAT3 across cancer types points to the possibility of using it as a common mediator of the anti-cancer activity of cucurbitacin.

A comparison of the studies summarized in Table 1 and Table 2 reveals considerable variation in the effective concentrations of cucurbitacin derivatives across cancer models. However, direct comparison of potency among studies remains difficult because of differences in cancer cell types, treatment duration, and the inconsistent reporting of IC50 values.

Cucurbitacins continued to be the major mode of action for killing cells by apoptosis. Most studies reported the activation of intrinsic mitochondrial apoptosis, which involved an increased Bax/Bcl-2 ratio, cytochrome c release, caspase activation, and PARP cleavage. However recent evidence has shown that cucurbitacins can also activate non-apoptotic cell death pathways. CuB activated the gasdermin-mediated signaling pathways to promote pyroptosis in lung and OSCC cells and disrupt iron homeostasis and antioxidant defense to promote ferroptosis in NSCLC cells and breast cancer cells. In conclusion, these results indicate that cucurbitacins can overcome apoptosis resistance by inducing other types of programmed cell death. The modulation of oxidative stress was another common finding. Increased ROS generation and decreased antioxidant systems, such as GSH, GPX4, Nrf2, and HO-1, have been reported in many studies. Increased ROS levels are responsible for mitochondrial dysfunction, DNA damage, ER stress, ferroptosis, and apoptosis. This suggests that alterations in redox homeostasis are a universal occurrence in the early stages of the multiple anti-cancer activities of cucurbitacins. Moreover, cucurbitacins target proteins such as FAK, F actin, vinculin and vinculin disrupting the cytoskeletal organization. Cucurbitacins inhibit metastasis, invasion, proliferation and angiogenesis of cancer cells by targeting MMPs, VEGF and FAK. Importantly, this review also focuses on the synergistic role of cucurbitacins with already established chemotherapeutics such as cisplatin, DOX, IM, gefitinib, paclitaxel, MTX and 5-FU. The combination exhibited significantly higher therapeutic efficiency in cancer cells compared to monotherapy. The process of reversal of chemoresistance includes the inhibition of STAT3, the downregulation of p-gp, cancer stem cell factors and the activation of apoptosis, suggesting the potential role of cucurbitacins as a chemosensitizing agent.

Despite their significant anti-cancer activity, clinical studies on cucurbitacins remain limited due to pharmacokinetic profiles, toxicity, and bioavailability-related challenges. For cucurbitacins, compounds such as CuB and CuIIa have a complete pharmacokinetic profile, whereas compounds such as CuE, CuD, and CuI lack sufficient data. Cucurbitacins exhibit poor water solubility, low bioavailability, rapid but incomplete absorption, and high distribution to organs such as the lungs, spleen, and kidney [124]. A pharmacokinetic study suggests that cucurbitacins are rapidly eliminated from the body, most probably due to biotransformation before excretion [125]. However, further pharmacokinetic studies must be conducted to determine the metabolism, plasma half-life, and excretion of cucurbitacins to support their clinical development.

Moreover, toxicity is another limitation associated with the use of cucurbitacins. According to some studies on toxicity, cucurbitacins appear to be toxic, even in low dosages on animals, while the dosages for the cure are extremely near to those that may cause toxicity in normal cells. Toxicity reports have concluded that CuE, CuD, CuC, and CuI are lethal to normal cells [19]. These challenges continue to restrict the translation of cucurbitacins into clinical studies, highlighting the need for further research focusing on pharmacokinetic properties, safety, and the development of drug delivery systems.

## 5. Conclusions

Cucurbitacins stand out as promising anti-tumor agents that can modulate multiple pathways involved in cancer proliferation and survival. This review emphasizes the importance of cucurbitacins in inducing cell-cycle arrest and programmed cell death such as apoptosis, autophagy, ferroptosis and pyroptosis in several cell lines and tumor models of lung, gastric, prostate, ovary, liver, breast, colorectal, pancreatic, leukemia, lymphoma, melanoma, glioblastoma, oral and osteosarcoma cancers by targeting several pathways and receptors like JAK/STAT, MAPK, Nrf2, PI3K/AKT/mTOR, CIP2A/PP2A/mTORC1, Notch, EGFR, HER2, VEGF, TFAP4, Wnt/ß-Catenin. They exhibit advantages over other compounds because of their ability to overcome drug resistance, improve the TME, and inhibit invasion and metastasis. This review does not highlight the activity of the rest of the cucurbitacin molecules, and other biological activities of cucurbitacins, such as anti-diabetic, anti-viral, and anti-parasitic activities. Future research should focus on establishing comprehensive structure–activity relationships among different cucurbitacin derivatives to identify the molecular features responsible for pathway selectivity. Future research must include a more detailed study on pharmacokinetic factors, such as the distribution, metabolism, stability and bioavailability of molecules over different cancers. Further clinical studies are needed to assess the safety, efficacy and therapeutic potential of cucurbitacins for the treatment of different cancers. Furthermore, future research should focus on combining cucurbitacins with traditional chemotherapeutic drugs, in vivo validation, and the development of drug delivery systems to boost drug bioavailability, increase drug uptake, and optimize therapeutic results. If we overcome these challenges, we may be able to incorporate cucurbitacin molecules into drugs and effectively cure cancer with minimal side effects.

## Figures and Tables

**Table 1:** Overview of cucurbitacins in combination with anti-cancer drugs to chemosensitize cancer cells.

Cucurbitacin Molecule (Concentration, Wherever Applicable)	Combinatorial Drug (Concentration)	Cancer	Pathway Targeted	Reference
CuE (In vitro 60 nM 100 nM) (In vivo 0.3 mg/kg)	Doxorubicin (In vitro 0–10 µM 500 nM) (In vivo 2 mg/kg)	Gastric (NCI-N87) (Female BALB/c nude mice)	Inhibits AKT pathway. Activates apoptosis.	[23]
CuE (In vitro 0.5 µM)	Myricetin (In vitro 20 µM)	Lung (A549 cells)	Targets the PI3K/AKT/mTOR pathway. Significant increase in apoptosis rate and cell-cycle arrest.	[24]
CuB (In vitro 10 µM)	Imatinib mesylate (In vitro 20 µM)	Colorectal (SW480) Breast (MCF-7)	Inhibits cell proliferation. Activates apoptosis. Induces S phase cell-cycle arrest. Decrease in *MMP-2*. Suppressing metastasis and invasion.	[25]
CuB (liposome encapsulation)	Doxorubicin (In vivo 3 mg/kg)	Liver (Hepa1-6, L02) (Male BALB/C nude mice)	Inhibits angiogenesis, migration, and invasion in tumor sites. Activates apoptosis.	[26]
CuE (In vitro 5 µM)	Sorafenib (In vitro 5 µM)	Liver (HepG2)	Regulates MAPK, STAT3 and PI3K/AKT/mTOR pathways. Induced apoptosis and cell-cycle arrest by inhibiting these pathways.	[27]
CuIIa (In vitro 2.86 µM)	Doxorubicin (In vitro 0.29 µM) (In vivo 3 mg/kg)	Liver (HepG2, Hep3B, H22) (Male ICR mice)	Induces apoptosis and immunogenic cell death. Regulates TME.	[28]
CuB (In vitro 0.1 µM)	Erastin (In vitro 1 µM, 10 µM)	Breast (MCF-7, MDA-MB-231)	Disrupts redox balance Activates Ferroptosis	[29]
CuB (In vitro 0.3 µM) (In vivo 0.5 mg/kg)	SCH772984 (ERK1/2 inhibitor) (In vitro 2 µM) (In vivo 25 mg/kg)	Pancreatic (BxPC-3, HPAC) (Female BALB/c nude mice)	Downregulates EGFR, STAT3, AKT, and S6 levels. Induces apoptosis.	[30]
CuB (In vitro 0.5 µM 40 µM)	Gefitinib (In vitro 10 µM)	Colorectal (HT-29, HCT-116)	Reduces pEGFR and pSTAT3 levels. Promotes apoptosis and cell-cycle arrest.	[31]
CuD (In vitro 0.5 µg/mL)	Doxorubicin (In vitro 1 µM)	Breast (MCF7)	Targets NF-kB signaling pathway. G2/M cell-cycle arrest and apoptosis.	[32]
CuB (In vitro 0.75 µg/mL 5 µg/mL)	Methotrexate (In vitro 0.75 µg/mL 5 µg/mL) (In vivo 5 mg/kg)	Osteosarcoma (U-2 OS) (BALB/C, nude mice)	Induces G2/M cell-cycle arrest and apoptosis.	[33]
CuE (In vitro 1 µM) (In vivo 5 mg/kg)	5-FU (In vitro 80 µM) (In vivo 25 mg/kg)	Colorectal (DLD1 and HCT-116) (Female nude mice)	Downregulates TFAP4, ß-Catenin, ABCC1 and MDR1 levels. Inhibition of tumor growth.	[34]
CuI (In vitro 100 nM)	5-FU	Colorectal (COLO205)	Suppression of STAT3. Decrease in MMP-9. Inhibition of cell invasion and proliferation.	[35]
CuB (In vitro 2 µM)	Cisplatin (In vitro 5 µM, 10 µM, 20 µM, 40 µM)	Ovarian (A2780)	Inhibition of NF-kB. Downregulation of pSTAT3 and Bcl-2. Activation of caspase and the apoptosis pathway.	[36]

Abbrevations: Protein kinase B (PKB also known as AKT); Phosphoinositide 3-Kinase (PI3K); mammalian Target of Rapamycin (mTOR); matrix metalloproteinase (MMP); Mitogen-Activated Protein Kinase (MAPK); Signal Transducer and Activator of Transcription 3 (STAT3); tumor microenvironment (TME); epidermal growth factor receptor (EGFR); Ribosomal Protein S6 (S6); Nuclear factor kappa-light chain enhancer of activated B cells (NF-kB); Transcription factor AP-4 (TFAP4); ATP-binding cassette subfamily C member 1 (ABCC1); multidrug resistance protein 1(MDR1); B cell lymphoma 2 (Bcl-2).

**Table 2:** Summary of cucurbitacin compounds reported to reverse chemoresistance in multiple cancer cells.

Cucurbitacin Molecule (Concentration)	Drug (Resistance Concentration, Wherever Applicable)	Cancer Model Used	Mechanism of Reversing Cancer Resistance	Reference
CuB (In vitro 80 nM, 120 nM)	Cisplatin (IC50 37.78 µM)	Gastric (SGC7901)	Downregulates CIP2A expression. Induces apoptosis and autophagy. Inhibits mTORC1 pathways.	[37]
CuB (In vitro 1 µM)	Paclitaxel (IC50 > 10 µM)	Ovarian (A2780)	Suppresses P-gp expression. Downregulates anti-apoptotic proteins. Activates apoptosis.	[38]
CuB (In vitro 0 µM, 0.04 µM, 0.2 µM, 1 µM, 5 µM) (In vivo 1 mg/kg)	Cisplatin	Ovarian (A2780) (BALB/C nude mice)	Activates cGAS. Induces DNA damage Suppresses p-mTOR levels. Inhibition of cell proliferation. Activation of apoptosis.	[39]
CuB (In vitro 143.2 µM 89.5 µM) (In vivo 10 mg/kg)	5-FU (IC25 400.5 µM)	Liver (BEL7402) (Male BALB/C nude mice)	Downregulates P-gp. Elevates cleaved caspase-3. Activates apoptosis.	[40]
CuI (In vitro 50 nM, 200 nM) (In vivo 0.5 mg/kg, 1 mg/kg)	Doxorubicin	Leukemia (JE6.1) Lymphoma (K-562)	Suppresses pSTAT3 levels. Downregulate Bcl-2. Induces apoptosis. Inhibits migration and proliferation of cells.	[41]
CuD (In vitro 0.5 µg/mL)	Doxorubicin 1 µM	Breast (MCF7)	Represses pSTAT3 expression.	[32]
CuE (In vitro 1 µM)	5-FU	Colorectal (HCT-8, HCT-116) Female nude mice	Downregulates ß-Catenin, ABCC1 and MDR1.	[34]
CuI (In vitro 100 nM)	5-FU	Colorectal (COLO205)	Suppresses STAT3 activation.	[35]
CuB (In vitro 2 µM)	Cisplatin (IC50 40 ± 2.5 µM)	Ovarian (A2780)	Reduces antioxidants, Dyrk 1B. Elevates ROS levels.	[36]

Abbreviations: Cancerous Inhibitor of Protein Phosphatase 2A (CIP2A); Mammalian Target of Rapamycin Complex 1 (mTORC1); P glycoprotein (P-gp); cyclic GMP-AMP synthase (cGAS); Signal Transducer and Activator of Transcription 3 (STAT3); B cell lymphoma (Bcl-2); ATP-binding cassette subfamily C member 1 (ABCC1); multidrug resistance protein 1 (MDR1); Dual-specificity tyrosine phosphorylation-regulated kinase 1B (Dyrk 1B); reactive oxygen species (ROS).

## Data Availability

No new research findings were generated in this study. All data presented in this study are included in the article.
